# Ipilimumab, a Promising Immunotherapy with Increased Overall Survival in Metastatic Melanoma?

**DOI:** 10.1155/2012/182157

**Published:** 2011-10-23

**Authors:** Gérald E. Piérard, François Aubin, Philippe Humbert

**Affiliations:** ^1^Department of Dermatopathology, University Hospital of Liège, 4000 Lièg, Belgium; ^2^Department of Dermatology, University Hospital of Besançon, 25000 Besançon, France; ^3^University of Franche-Comté, 25000 Besançon, France; ^4^EA3181, IFR133, 25000 Besançon, France; ^5^Inserm U645, IFR133, 25000 Besançon, France

## Abstract

Malignant melanoma (MM) is one of the most aggressive skin cancer. The therapeutic options remain limited for advanced MM, and those directed to the neoplastic cells have not brought major survival advantage so far. Immunotherapy is another targeted option. Ipilimumab, a monoclonal antibody directed to CTLA-4 present on cytotoxic T cells boosts immunity, particularly its anti-MM activity. Under treatment, the overall survival of patients with MM metastases is moderately but significantly increased. The immuno-related adverse effects may be severe and life threatening.

## 1. Introduction

Malignant melanoma (MM) is one of the most difficult neoplasms to treat. Unlike most common cancers, the last 20 years have seen no real improvement in systemic therapy for patients with metastatic MM. Currently the median survival for patients with metastatic MM is commonly limited to 6–9 months [1, 2]. However, some clinical and histopathological evidence exists providing clear demonstration of the ability of immunotherapy to mediate regression from the early to the advanced stages of MM [3–5]. The anti-MM lymphocytes consist of CD4+ and CD8+ T cells in part presenting as tumour-infiltrating lymphocytes (TIL). Among them, various clones of cytotoxic T lymphocytes (CTLs) are key cells to a specific anti-MM response [6, 7].

The past decade has witnessed much advances in the understanding of the complexity and redundancy in the immunological and other biological systems involved in MM proliferation, invasion, and metastasis [8–11]. A range of new drugs have been developed to specifically target the relevant pathways [12]. A number of immunotherapy trials for metastatic MM including cytokines, vaccines, adoptive immunotherapy, and their combinations were conducted in recent years [13]. A new promising MM therapy is emerging in the field of antibody-based specific targeting [14, 15]. Attempts were made at targeting either MM cells directly or immune cells involved in the anti-MM activity.

## 2. CTLA-4

The cytotoxic T-lymphocyte antigen 4 (CTLA-4) corresponding to CD152 is expressed at the surface of some T cells. CTLA-4 is a member of the immunoglobulin superfamily [13–15] acting as a downregulator of the immune system and playing a key role in the inhibition of the anticancer immunity [16]. With the exception of CD4+CD25+, Foxp3+ T-regulatory cells (Tregs) resting lymphocytes do not express CTLA-4. Stimulation of CTLA-4 at the CTL surface results in inhibition of their proliferation. 

The CTLA-4 expressed at the CTLs surface binds to both B7-1 and B7-2 (CD80 and CD86) ligand pairs present on antigen-presenting cells. Their interaction activates a cell-signaling cascade leading to the cell cycle arrest of CTLs [16]. This mechanism results in T-cell anergy and interferes with both IL-2 secretion and IL-2 receptor expression, leading in turn to inhibition of T-cell priming and immune escape, thereby allowing neoplastic growth [17]. It produces immune control by inhibition of T-cell responses contributing to self-antigen tolerance [18]. By contrast, CD28 binding to B7-1 and B7-2 leads to stimulation of CTL proliferation and production of IL-2. Because of its expression on dendritic cells, effector T cells, and regulatory T cells, CTLA-4 exhibits multiple roles at various stages of the immune response. 

Blocking CTLA-4 is thought to shift the balance of the immune response, enhancing both the recognition of tumour antigens and the neoplastic regression [17]. Consequently, this process decreases tolerance to self-antigens, leading to autoimmunity [5, 18]. CTLA-4 blockade causes a dynamic shift in the ratio between Tregs and CD8+ TCLs culminating in effective immune recognition of neoplasms [19]. This event was documented in posttreatment biopsies of neoplasms treated with CTLA-4 blockade and correlated with therapy-mediated tumoral necrosis [20, 21].

## 3. Tremelimumab

Tremelimumab was a fully human IgG2 anti-CTLA-4 monoclonal antibody. Partial response (PR) was initially reported to reach 6.6% for a period extending 8.9 to 29.8 months [22, 23]. Despite such early promise, a more recent phase III trial failed to show a greater survival benefit than traditional chemotherapy. The drug was subsequently abandoned.

## 4. Ipilimumab

Ipilimumab (MDX-010: Medarex, Bristol-Myers Squibb) is a fully humanized IgG1 monoclonal antibody directed to CTLA-4 [13–15, 19–21, 24–29]. Objective response rates combining complete response (CR) and PR were in the range of 5–20% [15, 29]. Disease control rates (CR + PR + stable disease) were reported averaging 15–30%. In contrast, the two therapies approved by the FDA, high-dose interleukin-2, and dacarbazine are each associated with response rate of only 10 to 20% and a small percentage of CR. They are not thought to improve overall survival (OS) [30].

Studies involving higher doses of ipilimumab were associated with higher response rates but with increased toxicities [19, 24–26]. Ipilimumab is the first therapeutic agent showing prolonged OS (median OS: 10.1 months) in patients with metastatic MM. This figure must be compared to the median survival in such a patient population with other current therapies generally reaching 6–9 months [1, 2, 28].

Rates of adverse reactions to ipilimumab, particularly autoimmune events, appear to be dose- and schedule-dependent. Toxicities associated with ipilimumab differ from those typically related to regular cytotoxic chemotherapy [31–33], and they create unique challenges in diagnosis and clinical management [34]. The majority of adverse events to ipilimumab are immune mediated, corresponding to the so-called “immune-related adverse events (irAEs)” [35, 36]. The irAEs affect a range of organs, including the skin, gastrointestinal tract, and endocrine glands. Antinuclear antibodies (ANA) are not associated with irAEs, and they have no diagnostic value in this setting, since many patients with MM show baseline elevations of ANA titers.

The ipilimumab irAEs are dose-dependent, schedule-related, and cumulative [34]. Grade 3 and 4 irAE were reported in 20–30% of patients. Close clinical and laboratory monitoring is required for early detection and timely initiation of treatment with immunosuppressive therapies. Most irAEs were manageable and generally reversible under corticotherapy. Long-term residual irAEs requiring treatment were reported at 2-year followup in phase III trials, primarily corresponding to dermatologic effects (rash, vitiligo, and pruritus), colitis/diarrhea, and endocrine-related adverse events [25]. In addition, life-threatening irAEs (bowel perforation due to immune colitis) and treatment-related mortality were reported in about 2% of ipilimumab-treated patients. Additional immunosuppression was sometimes required [37, 38]. Up to 50% of treatment-related deaths were associated with irAEs [29].

## 5. Conclusion

Immunotherapy, particularly blockade of the CTLA-4 pathway, has already proven an effect against advanced MM. Ipilimumab is the first agent demonstrating promise in the treatment of metastatic MM. The positive but modest OS benefit requires more investigations. It seems essential to tailor treatment options to those patients most likely to benefit, especially because the treatment is associated with frequent and sometimes life-threatening irAE. Overall, the inherent risks of immunotherapy require judicious use in appropriately selected patients by well-informed clinicians and patients.

## References

[1] Middleton MR, Grob JJ, Aaronson N (2000). Randomized phase III study of temozolomide versus dacarbazine in the treatment of patients with advanced metastatic malignant melanoma. *Journal of Clinical Oncology*.

[2] Bedikian AY, Millward M, Pehamberger H (2006). Bcl-2 antisense (oblimersen sodium) plus dacarbazine in patients with advanced melanoma: the oblimersen melanoma study group. *Journal of Clinical Oncology*.

[3] Henry C, Lapière M, Franchimont C, Piérard GE, Lapière CM, Ackerman AB (1981). Immunotherapy by dinitrochlorobenzene of melanomas of the skin. I- Methods, clinical effects and survival rates. *Pathology of Malignant Melanoma*.

[4] Piérard GE, Henry C, Franchimont C, Lapière M, Ackerman AB, Lapière CM, Ackerman AB (1981). Immunotherapy by dinitrochlorobenzene of melanomas of the skin. II- Histology of the cytotoxic effect. *Pathology of Malignant Melanoma*.

[5] Dudley ME, Wunderlich JR, Yang JC (2005). Adoptive cell transfer therapy following non-myeloablative but lymphodepleting chemotherapy for the treatment of patients with refractory metastatic melanoma. *Journal of Clinical Oncology*.

[6] Komenaka I, Hoerig H, Kaufman HL (2004). Immunotherapy for melanoma. *Clinics in Dermatology*.

[7] Klein O, Ebert LM, Nicholaou T (2009). Melan-a-specific cytotoxic T cells are associated with tumor regression and autoimmunity following treatment with anti-CTLA-4. *Clinical Cancer Research*.

[8] Quatresooz P, Pierard GE, Pierard-Franchimont C (2009). Molecular pathways supporting the proliferation staging of malignant melanoma. *International Journal of Molecular Medicine*.

[9] Quatresooz P, Pierard-Franchimont C, Paquet P, Pierard GE (2010). Angiogenic fast-growing melanomas and their micrometastases. *European Journal of Dermatology*.

[10] Quatresooz P, Piérard GE (2011). Malignant melanoma: from cell kinetics to micrometastases. *American Journal of Clinical Dermatology*.

[11] Quatresooz P, Reginster M-A, Piérard GE (2011). 'Malignant melanoma microecosystem': immunohistopathological insights into the stromal cell phenotype a review. *Experimental and Therapeutic Medicine*.

[12] Lorigan P, Eisen T, Hauschild A (2008). Systemic therapy for metastatic malignant melanoma - From deeply disappointing to bright future?. *Experimental Dermatology*.

[13] Sznol M (2009). Betting on immunotherapy for melanoma. *Current Oncology Reports*.

[14] Piérard-Franchimont C, Quatresooz P, Paquet P, Nikkels AF, Piérard GE (2009). A range of targeted treatments using monoclonal antibodies in dermatology. Current and future practice. *Revue Médicale de Liège*.

[15] Hodi FS, O’Day SJ, McDermott DF (2010). Improved survival with ipilimumab in patients with metastatic melanoma. *New England Journal of Medicine*.

[16] Korman AJ, Peggs KS, Allison JP (2006). Checkpoint blockade in cancer immunotherapy. *Advances in Immunology*.

[17] Gajewski TF (2010). Improved melanoma survival at last! Ipilimumab and a paradigm shift for immunotherapy. *Pigment Cell and Melanoma Research*.

[18] Laurent S, Carrega P, Saverino D (2010). CTLA-4 is expressed by human monocyte-derived dendritic cells and regulates their functions. *Human Immunology*.

[19] Ku GY, Yuan J, Page DB (2010). Single-institution experience with ipilimumab in advanced melanoma patients in the compassionate use setting lymphocyte count after 2 doses correlates with survival. *Cancer*.

[20] Hodi FS, Butler M, Oble DA (2008). Immunologic and clinical effects of antibody blockade of cytotoxic T lymphocyte-associated antigen 4 in previously vaccinated cancer patients. *Proceedings of the National Academy of Sciences of the United States of America*.

[21] Hodi FS, Oble DA, Drappatz J (2008). CTLA-4 blockage with ipilimumab induces significant clinical benefit in a female with melanoma metastases to the CNS. *Proceedings of the National Academy of Sciences*.

[22] Kirkwood JM, Lorigan P, Hersey P (2010). Phase II trial of tremelimumab (CP-675,206) in patients with advanced refractory or relapsed melanoma. *Clinical Cancer Research*.

[23] Camacho LH, Antonia S, Sosman J (2009). Phase I/II trial of tremelimumab in patients with metastatic melanoma. *Journal of Clinical Oncology*.

[24] Maker AV, Yang JC, Sherry RM (2006). Intrapatient dose escalation of anti-CTLA-4 antibody in patients with metastatic melanoma. *Journal of Immunotherapy*.

[25] Downey SG, Klapper JA, Smith FO (2007). Prognostic factors related to clinical response in patients with metastatic melanoma treated by CTL-associated antigen-4 blockade. *Clinical Cancer Research*.

[26] Weber JS, O’Day S, Urba W (2008). Phase I/II study of ipilimumab for patients with metastatic melanoma. *Journal of Clinical Oncology*.

[27] Wolchok JD, Neyns B, Linette G (2010). Ipilimumab monotherapy in patients with pretreated advanced melanoma: a randomised, double-blind, multicentre, phase 2, dose-ranging study. *The Lancet Oncology*.

[28] O’Day SJ, Maio M, Chiarion-Sileni V (2010). Efficacy and safety of ipilimumab monotherapy in patients with pretreated advanced melanoma: a multicenter single-arm phase II study. *Annals of Oncology*.

[29] Thumar JR, Kluger HM (2010). Ipilimumab: a promising immunotherapy for melanoma. *Oncology*.

[30] Atkins MB, Lotze MT, Dutcher JP (1999). High-dose recombinant interleukin 2 therapy for patients with metastatic melanoma: analysis of 270 patients treated between 1985 and 1993. *Journal of Clinical Oncology*.

[31] Guillot B, Bessis D, Dereure O (2004). Mucocutaneous side effects of antineoplastic chemotherapy. *Expert Opinion on Drug Safety*.

[32] Wyatt AJ, Leonard GD, Sachs DL (2006). Cutaneous reactions to chemotherapy and their management. *American Journal of Clinical Dermatology*.

[33] Piérard GE, Paquet P, Piérard-Franchimont C, Rorive A, Quatresooz P (2007). Cutaneous adverse reactions to chemotherapy and their management. *Revue Medicale de Liege*.

[34] Lowe M, Delman KA (2010). Ipilimumab for advanced melanoma: let's not throw caution to the winds. *Oncology*.

[35] Phan GQ, Yang JC, Sherry RM (2003). Cancer regression and autoimmunity induced by cytotoxic T lymphocyte-associated antigen 4 blockade in patients with metastatic melanoma. *Proceedings of the National Academy of Sciences of the United States of America*.

[36] Atria P, Phan GQ, Maker AV (2005). Autoimmunity correlates with tumor regression in patients with metastatic melanoma treated with anti-cytotoxic T-lymphocyte antigen-4. *Journal of Clinical Oncology*.

[37] Weber J (2009). Ipilimumab: controversies in its development, utility and autoimmune adverse events. *Cancer Immunology, Immunotherapy*.

[38] Minor DR, Chin K, Kashani-Sabet M (2009). Infliximab in the treatment of anti-CTLA4 antibody (ipilimumab) induced immune-related colitis. *Cancer Biotherapy and Radiopharmaceuticals*.

